# Mobile Health Technologies May Be Acceptable Tools for Providing Social Support to Tuberculosis Patients in Rural Uganda: A Parallel Mixed-Method Study

**DOI:** 10.1155/2020/7401045

**Published:** 2020-01-07

**Authors:** Angella Musiimenta, Wilson Tumuhimbise, Esther C. Atukunda, Aaron T. Mugaba, Conrad Muzoora, Mari Armstrong-Hough, David Bangsberg, J. Lucian Davis, Jessica E. Haberer

**Affiliations:** ^1^Mbarara University of Science and Technology, Mbarara, Uganda; ^2^Angels Compassion Research and Innovations Centre, Mbarara, Uganda; ^3^Uganda Tuberculosis Implementation Research Consortium, Makerere University, Kampala, Uganda; ^4^Department of Epidemiology of Microbial Diseases, Yale School of Public Health, New Haven, Connecticut, USA; ^5^Pulmonary, Critical Care and Sleep Medicine, Yale School of Medicine, New Haven, Connecticut, USA; ^6^New York University College of Global Public Health, New York, New York, USA; ^7^Oregon Health & Science University-Portland State University School of Public Health, USA; ^8^Harvard Medical School, Boston, MA, USA; ^9^Massachusetts General Hospital Center for Global Health, Boston, MA, USA

## Abstract

**Background:**

Social support has been shown to mitigate social barriers to medication adherence and improve tuberculosis (TB) treatment success rates. The use of mobile technology to activate social support systems among TB patients, however, has not been well explored. Moreover, studies that tie supportive SMS (Short Message Service) texts to electronic monitoring of TB medication adherence are lacking.

**Objective:**

To explore TB patients' current access to social support and perceptions of utilizing real-time adherence monitoring interventions to support medication adherence.

**Methods:**

We purposively selected TB patients who owned phones, had been taking TB medications for ≥1 month, were receiving their treatment from Mbarara Regional Referral Hospital, and reported having ≥1 social supporter. We interviewed these patients and their social supporters about their access to and perceptions of social support. We used STATA 13 to describe participants' sociodemographic and social support characteristics. Qualitative data were analyzed using content analysis to derive categories describing accessibility and perceptions.

**Results:**

TB patients report requesting and receiving a variety of different forms of social support, including instrumental (e.g., money for transport and other needs and medication reminders), emotional (e.g., adherence counselling), and informational (e.g., medication side effects) support through mobile phones. Participants felt that SMS notifications may motivate medication adherence by creating a personal sense of obligation to take medications regularly. Participants anticipated that limited financial resources and relationship dynamics could constrain the provision of social support especially when patients and social supporters are not oriented about their expectations.

**Conclusion:**

Mobile telephones could provide alternative approaches to providing social support for TB medication adherence especially where patients do not stay close to their social supporters. Further efforts should focus on optimized designs of mobile phone-based applications for providing social support to TB patients and training of TB patients and social supporters to match their expectations.

## 1. Introduction

Globally, Uganda ranks among the 30 countries with the highest burden of TB (tuberculosis), with a prevalence rate of 253/100,000 people and an incidence rate of 201/100,000 people [1]. Although treatment is freely available in Uganda, significant treatment adherence challenges remain, constraining TB treatment success and increasing its transmission. The implementation of DOTS (Direct Observed Treatment Short Course) has been limited in Uganda. This is because DOTS demands significant time and financial burden from patients as they travel to the clinic; it also demands time commitment from health workers and treatment supporters who have to supervise patients taking their medication [2]. Social factors such as poverty, stigma, and limited knowledge of tuberculosis (TB) can constrain adherence to tuberculosis medications [3–8]. Provision of social support, defined as instrumental, emotional, and informational assistance from one's social network, can enhance TB medication adherence by mitigating the social barriers to medication adherence. For instance, money can facilitate transport to the clinic for pill refills, while encouragement or positive feedback makes people feel cared for and empowered to cope with stigma and social discrimination [9, 10]. Importantly, social support has been shown to improve clinical outcomes, such as the treatment success rate [11].

Traditional approaches to providing social support (such as face-to-face counselling interventions) may be limited by geographical boundaries and are often expensive due to the time and transport costs involved. Given the widespread adoption of wireless technologies, such as mobile phones in sub-Saharan Africa (76% ownership in 2015; [12]), these technologies can provide accessible and potentially affordable means of providing social support. In a recent study carried out in central Uganda, 75% of TB patients reported owning mobile phones and being willing to receive TB-related communication via their phones [13]. Mobile phones are thus a promising alternative approach for providing social support to TB patients.

Prior studies have utilized mobile technology to support adherence to TB medications, including randomized, controlled trials of SMS reminders in Pakistan [14], daily mobile phone calls in Thailand [15], and video directly observed therapy (VDOT) in California and Mexico [16]. These approaches have generally been found feasible and acceptable [16] and have also shown improvements in clinical outcomes, including treatment completion and sputum conversion rates in some studies (e.g., [15]) but not all (e.g., [17]). Overall, mobile technology is becoming widely used to support TB adherence, although the majority of studies have focused on reminders and DOT (directly observed therapy). Moreover, the use of mobile technologies to activate social support systems has not been well explored. Moreover, there is lack of studies that tie supportive SMS texts to electronic monitoring (which may be an attractive alternative to DOT) although limited evidence in HIV is promising [18–20].

In this study, we used a parallel mixed-method design to explore the types of social support that adults with presumed drug-sensitive TB in rural Uganda receive during TB treatment and to assess how patients utilize mobile phones to obtain social support. We also explored how these adults would perceive social support for a possible future TB adherence intervention that we were planning. This intervention includes (1) real-time audits of adherence using a digital monitoring device, (2) SMS reminders to patients, and (3) SMS notifications to patients' social supporters. WHO [21] guidelines recommend directly observed therapy (DOT) using a community-based model in which a family member or close friend is designated as an adherence supporter, which includes the responsibility to watch the patient take his or her medications every day. In this study, we define social supporters as friends or family who have previously helped TB patients with medication or other needs (such as transport to clinic, food, child care, and/or guidance).

## 2. Materials and Methods

### 2.1. Study Design and Setting

This formative study employed a parallel mixed-method study design that utilized semistructured interviews and surveys. TB patients were recruited from the TB clinic within Mbarara Regional Referral Hospital (MRRH) in rural, southwestern Uganda. Uganda ranks among the 30 countries with the highest burden of TB/HIV in the world in 2017 [22]. The treatment completion rate stands at 75% in Uganda [23]. The Mbarara TB unit provides care to approximately 600 TB patients annually. TB treatment to newly diagnosed patients is provided according to WHO guidelines [21] with a daily short course regimen delivered in fixed-dose combination tablets containing isoniazid, rifampin, pyrazinamide, and ethambutol for two months. At the two-month visit, sputum is examined, and those who convert the smear examinations to negative continue with isoniazid and rifampin only for four months in the continuation phase. The period of treatment can be extended up to eight months to cater for missed medication pick-ups. All patients with new cases of TB self-administer their medications. The clinic does not employ directly observed therapy due to inadequate staff time and excessive travel costs for patients to attend the clinic.

### 2.2. Selection of Study Participants

Between June 2017 and June 2018, authors AMT and WT purposively selected patients receiving TB treatment in Mbarara. We aimed to achieve balanced representation by gender and HIV status to elicit diverse perspectives. Inclusion criteria for TB patient participants were as follows: (a) having documented drug-sensitive TB, (b) receiving treatment with a first-line 6-month course of anti-TB regimen as described above for at least one month, (c) having a personal mobile phone, (d) able to send and receive SMS, (e) being 18 years or older, (f) residing in Mbarara district, (g) being willing and able to give consent, (h) being willing and able to name one social supporter, and (i) being able to speak the local language (Runyankole) or English.

We asked patients to identify social supporters at enrollment on the basis of an ongoing relationship, social supporter knowledge of the patient's TB status, and previous or current provision of social support (e.g., assistance to travel to the clinic and/or medication adherence advice) to the study participant. Inclusion criteria for the social supporter participants were as follows: (a) owning a personal mobile phone, (b) being able to send and received SMS, (c) being 18 years or older, (d) residing in Mbarara district, (e) knowing the study participant's TB/HIV status (ascertained through patients' self-reports), and (f) willing and able to provide consent.

### 2.3. Study Procedures

#### 2.3.1. Intervention Demonstration

**Figure 1:**
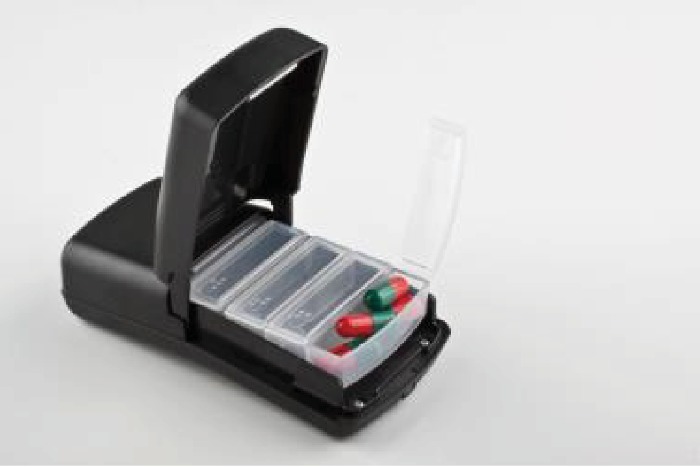
The Wisepill device.

At enrollment, and before conducting each interview, we oriented each participant about the planned interventions to support adherence to TB medication. First, we explained that a real-time adherence monitor (Wisepill device; Wisepill Technologies, Cape Town, South Africa; Figure 1) is a pill bottle that records a date-and-time stamp when opened. We demonstrated how the adherence monitor works, including how to open it and put tablets in and how to close it after taking tablets. We indicated that the device monitors openings as proxies of medication ingestion and sends alerts to researchers if it is not opened by the expected time. We demonstrated how the monitor holds up to 28 tablets of TB medication. Participants were then given the monitor and asked to explain what it does and to practically demonstrate how it works to the researcher. We then explained to participants how we will send SMS reminders on a scheduled (e.g., daily) or triggered (e.g., triggered by a delayed/missed dose as detected through the monitor) basis to TB patients to help them take their medications. We further explained that the device could send SMS notifications of nonadherence to the patients' social supporters to encourage provision of assistance to the patient.

#### 2.3.2. Data Collection

Immediately after orienting participants about the intervention (at enrolment), authors WT and AMT administered surveys eliciting information about sociodemographics, health status, social support, and general preferences for the planned wireless intervention. Within two weeks of enrolment, WT, AM, and AMT carried out semistructured in-depth interviews with TB patients (following reorientation about the intervention); both authors are bilingual in English and Runyankole and trained in qualitative research and research ethics. All questions in the interview guide were translated into Runyankole and back-translated to English by a different translator. Interviews were conducted in Runyankole, digitally recorded, transcribed, and translated to English. Interviews were conducted in a private space at the research office near the MRRH. Each interview lasted between 30 and 60 minutes. We elicited information about any current social support obtained by TB patients and how they feel about the social support they obtain, as well as their expectations of the wireless adherence monitoring intervention vis-à-vis social support. In interviews with social supporters, we also solicited information about the challenges they faced in providing medication adherence-related social support to TB patients and how the intervention might influence these challenges. Interviews were conducted until thematic saturation was achieved. Following each interview, author AM, with support from JEH and JLD, reviewed the transcripts for quality, clarity, and detail.

#### 2.3.3. Analysis

Participants' sociodemographic details and preferences were summarized descriptively using STATA 13 by ATM and TW. We used inductive content analysis [24] to derive qualitative categories describing how participants currently use their phones to support TB-related treatment and how they perceived the planned wireless adherence monitoring intervention. Initially, AM, WT, and ATM reviewed and discussed 20% of transcripts for content relevant to current social support and expectations of the intervention. AM and WT then assembled a codebook from the identified concepts, using an iterative process, which included developing codes to represent content, writing operational definitions, and selecting illustrative quotes. JEH, JLD, and DB also reviewed and discussed the codebook. Differences in coding were harmonized through discussion. Following completion of the codebook, interviews were coded by AM and WT using NVIVO 11.

#### 2.3.4. Ethical Approval

Ethical approvals were obtained from the Research Ethics Committee of Mbarara University of Science and Technology, the Uganda National Council for Science and Technology, and the Partners Human Research Committee for Massachusetts General Hospital.

## 3. Results and Discussion

### 3.1. Participant Characteristics

Of 53 screened TB patients, 18 (34%) were excluded for the following reasons (individuals could have >1 criterion): having no cellphone (*n* = 6; 11.3%), inability to use SMS text messages (*n* = 5; 9.4%), unwillingness or inability to name at least one social supporter (*n* = 3; 5.7%), being <18 years old (*n* = 2; 3.8%), having drug-resistant TB (*n* = 1; 1.9%), and/or inability to provide informed consent (*n* = 1; 1.9%). A total of 35 TB patients, of whom 15 (42.8%) were persons living with HIV/AIDS, enrolled in the study between February 2017 and April 2018.

Of 24 screened social supporters, 15 (62.5%) were enrolled in the study. Nine social supporters were excluded for the following reasons (individuals could have >1 criterion): having no cellphone (*n* = 3; 12.5%), living beyond Mbarara district (*n* = 2; 8.33%), not knowing the study participant's TB/HIV status (*n* = 2; 8.3%), being <18 years of age (*n* = 1; 4.2%), and inability to provide informed consent (*n* = 1; 4.2%).

The sociodemographics, self-reported HIV status, and TB treatment progress of the study participants are indicated in Table 1. Just over half of the TB patients were male and the median age was 32 years. The mean period of TB treatment completed at the time of interview was 3.3 months. Most social supporters were female with a median age of 37 years. The majority of social supporters were TB patients' biological relatives (*n* = 8, 53%), 4 (27%) were spouses, and 3 (20%) were friends.

### 3.2. Survey Results

The most frequently reported type of social support was being reminded/encouraged to take medication—24 (69%) (Table 2). The majority of patients also reported that they were currently not receiving enough social support—21 (60%). Patients preferred SMS notifications that do not instruct how social supporters should help—20 (57.2%), compared to notifications that do offer such guidance.

### 3.3. Interview Results

TB patient participants reported utilizing mobile phones to obtain social support related to TB medication and treatment by (1) requesting and receiving instrumental support from social supporters, (2) requesting and receiving emotional support from social supporters, and (3) receiving informational support from healthcare providers. In reference to the anticipated intervention, social supporters and TB patients reported that SMS notifications may motivate medication adherence. Social supporters reported being concerned that they would at times be unable to provide the required support. TB patients reported the possibility of strain on relationships as a result of sending notifications linked to missed doses to social supporters. TB patients highlighted the need for the clinic/researchers to orient social supporters and patients about their expectations after receiving SMS notifications of nonadherence.

### 3.4. Use of Mobile Phones for Social Support

#### 3.4.1. Requesting and Receiving Instrumental Support

TB patients described having used mobile phones to call their social supporters to request and obtain financial assistance in the form of mobile money (i.e., money sent and received using mobile phones). Patients reported using the obtained financial assistance to transport themselves to the clinic to obtain TB medication, buy food and drinks to ease pill taking, and pay bills both related and unrelated to TB (e.g., clinic bills, children's school fees, and housing bills).

Interviewer (I): What challenges do you face in picking your TB medication from the hospital? For example, how do you normally come to the hospital to pick your TB medication?

Respondent (R): When I got sick, I moved to stayed with my sister so that she can assist me with washing, cooking, and generally taking care of me when I am too weak. So, I do not stay with my mother who provides money for transport to the clinic for my medication. I always have to use my mobile phone to call my mother to send me transport using mobile money. (Female, 39 yrs, TB patient living with HIV)

This support was particularly helpful for patients without a regular source of income.

#### 3.4.2. Requesting and Receiving Emotional Support

TB patients reported using mobile phones to call their social supporters, who in turn provided them with multiple forms of emotional social support. Common modes of emotional support included encouraging patients to take medication, encouraging them to live positively, and accompanying them to the TB clinic.

I: Please tell me about a time when someone used a mobile phone to help you with anything related to your TB?

R: I used my phone to call my sister, and she escorted me to the hospital, and took me to the theatre. She would encourage me to take medicine, and ensured that I was taken good care of myself.

I: How did you feel about the support that she gave to you?

R: I felt good and cared for because she is the one that was always with me.

I: So why do you think she helped you the way she helped you?

R: She helped because she cares about my life. (Female, 28 yrs, TB patient)

Patients described feeling good and cared for when their social supporters checked on them during telephone and SMS interactions.

#### 3.4.3. Informational Support from Healthcare Providers

In addition to their social networks, TB patients also reported using their mobile phones to call healthcare providers and inquire about side effects, such as feeling dizzy or experiencing a change of urine color after taking the medication. These calls helped patients get useful medication-related advice, such as the need to eat and drink before taking medication.

I: First, I would like to know a little about how you use your cell phone. Do you ever use your phone for anything related to your health?

R: Yes. Sometimes when I take my medication and become dizzy I call the nurse nearby our trading center called X and she tells me what to do.

I: Please tell me more about that.

R: When I call her, she tells me that it's the side effect of the TB pills and that to reduce it, I need to drink a lot and ensure that I do not take medicine on an empty stomach. (Female, 37 yrs, TB patient)

TB patients, especially those who stayed far from the clinic, reported calling healthcare providers to make inquiries about the availability of drugs and clinic opening times. This information saved patients the expense of having to buy medicine from private pharmacies when they are freely available at the public clinic or traveling to the clinic outside operating hours.

I: Have you ever gotten assistance in taking your TB medication through your mobile phone?

R: Yes I have ever called a doctor because I wanted to find out about the availability of the drugs at the TB treatment unit. The doctor told me that the drugs are always available and advised me to always come and pick the medication. He also discouraged me from buying medication from the pharmacy in case of failure to come to the hospital because they are expensive in terms of costs. (Male 39 yrs, TB patient)

Instrumental and emotional support from friends and family, as well as informational support from clinicians, are important resources that can be accessed using mobile phones and used to facilitate completion of TB treatment.

### 3.5. Perceptions about the Planned Intervention

#### 3.5.1. SMS Notifications to Social Supporters May Motivate TB Medication Adherence

Regarding the planned wireless adherence monitoring technology, social supporters and TB patients reported that receiving SMS texts when patients miss taking medication may “force” TB patients “not to forget taking medicine on time.” This “force” was described positively, because it is motivated by the shared desire for the patient to get well.

I: How do you think [study participant] will feel when he knows that you receive his SMS when he misses taking his medication?

R: He will like it very well and knowing that I will receive his SMS text when he misses taking his medication will force him not to forget taking his medicine on time.

I: Why would he be forced not to forget?

R: He knows me as a very strict person who has cared about his sickness since testing up to now. I agreed with him to always tell me, how he picks his medication and whenever he does medical checkup since it's me who gives him transport as my son. (Male, 65 years, social supporter)

I: How do you feel about sending SMS notification to your social supporter when you miss taking your pills in time?

R: It is a good thing because it will help me get cured quickly.

I: Please tell me more about that.

R: It forces me to take my drugs on time to get well, and also make him happy by seeing that he did not waste his efforts in supporting me. (Male, 45 years, patient)

Social supporters felt that access to updates about patients' adherence would create a personal sense of obligation to take medication on time among patients.

#### 3.5.2. Economic Constraints Could Limit Resources to Provide Support

Despite the anticipated motivation, participants had some concerns about implementing the planned intervention. First, although social supporters reported a desire and willingness to assist TB patients, those without a regular source of income reported being worried about the possibility of not being able to provide support for the TB patients even after receiving the SMS notification from the planned intervention.

It is not easy for me now, and it would not be easy for me when you send me a notification. I have to provide him all the support he needs to take his medicine well. Yet, I am also a poor woman struggling to live. (Female, 63, social supporter)

This inability to provide support when needed created feelings of disappointment and embarrassment for both the social supporters and TB patients.

#### 3.5.3. Receipt of the Planned SMS Notifications Could Strain Relations

Second, in addition to stress arising from limited resources, some TB patients and social supporters expressed concern that adherence-related messages could introduce conflict into their relationships. For example, patients noted that receiving notifications of missed doses could strain their relationship with social supporters.

“Once she knows that I have missed taking my medication and she has received an SMS notification from you. I am sure she will quarrel with me. Most of the times when she finds that I have forgotten taking my TB medication, she quarrels.” (Male, 50 yrs, TB patient)

This strain could potentially negatively affect the relationships between TB patients and their social supporters.

#### 3.5.4. Orienting Social Supporters and TB Patients May Improve the Impact of SMS Notifications

In response to these challenges, TB patients and social supporters described the need to be oriented about the planned SMS notifications. This orientation could be done by the clinic or researchers and should highlight times and/or scenarios when SMS notifications can be sent and multiple ways how social supporters can help. During these orientations, patients can be helped to understand that social supporters may at times be unable to provide instrumental support due to inadequate resources.

I: So do you want the SMS notifications to inform your social supporter that you forgot to swallow your TB medication in order for her to help you well in taking you are TB medication?

R: The best thing is to tell me to come with her here and you teach both of us so that each of us knows what to do after receiving the SMS. (Male, 50 yrs, TB patient)

Providing orientation may be helpful in matching expectations of TB patients and their social supporters.

## 4. Discussion

In this mixed-method study of social support for adherence to TB treatment, TB patients described requesting and receiving instrumental, emotional, and informational social support using their mobile phones. Reminders and encouragement were the most common type of support received, followed by transport to the clinic, food and drinks, and help with chores. Participants felt that SMS notifications may motivate medication adherence by creating a personal sense of obligation to take medication on time. However, TB patients and social supporters reported that limited resources and relationship dynamics may constrain the provision of social support. Participants expressed the need to be oriented about the use of SMS notifications before they start receiving them in order to match expectations of patients and those of social supporters.

Traditional approaches to providing social support (such as counselling support, family and community support, and health education) have been shown to positively impact TB treatment success [9, 10]. A systematic review by van Hoorn and colleagues (which includes studies from low income countries of Nepal, Burkina Faso, and Haiti) reports that social support in the form of counseling, food supplements, home visits, and economic support improves TB treatment completion [25]. Other studies [26, 27] report the positive influence of instrumental support (in the form of monetary incentives and food packages) on TB treatment success and TB medication adherence in Asia and Russia. A randomized, controlled trial carried out by Tola and colleagues reports decreased nonadherence to TB medication adherence as a result of a social support intervention composed of counselling and health education in Ethiopia [28]. Despite the benefits, traditional approaches for providing social support may involve transport and time burdens, especially among patients who do not stay close to their social supporters. Delivering social support using mobile phones may be an inexpensive, accessible, and convenient approach that can potentially overcome geographical barriers to the provision of social support.

In this study, TB patients and their supporters largely agreed that SMS reminders linked to adherence monitoring technology could improve adherence. Such interventions have been shown to increase adherence to HIV antiretroviral therapy. In China, mobile phone-based SMS reminders are reported to be acceptable and useful in establishing antiretroviral adherence routines [29]. In South Africa, adherence measured by a real-time monitoring technology emerged as one of the best predictors of antiretroviral drug resistance and virological failure [30]. In the same setting in Uganda, a mobile adherence monitoring intervention composed of real-time adherence technology and SMS reminders was reported to be acceptable [19] and to improve adherence to antiretroviral therapy [18]. Similar or related interventions need to be tested in supporting TB medication adherence.

Our findings emphasize the need for researchers and intervention designers to consider social support in light of complex interpersonal relationships. Sending SMS notifications to social supporters when patients miss their TB medications may motivate patients to consistently take their medication on time to avoid letting down the efforts of social supporters who are caring for their health. Social supporters' frequent receipt of SMS notifications may, however, be perceived as patients' lack of commitment to medication adherence. This scenario may potentially result in misunderstandings which may affect the quality of support provided and the relationship itself, as noted by our participants. Additionally, the anticipated conflict as a result of unmatched or unmet expectations between patients and their social supporters was indeed reported in the antiretroviral adherence intervention in the same setting [31] and no definitive benefit of social support was seen [18].

Since findings indicate that potential strain on relationships and resources could limit the feasibility/acceptability of social support interventions, managing such strains is likely critical to the successful implementation of these interventions. Mobile phone-based support has a great potential, but the complexities of resource scarcity and interpersonal relationships necessitate careful human management of the process. Additional interventions for boosting the economic status (e.g., [32]) of social supporters or identifying alternative sources of support when possible (e.g., other members of the patient's social network) may improve the feasibility of the social support intervention. Providing orientation and counseling to patients and social supporters could also help in clarifying their roles, managing and harmonizing expectations, and highlighting the negative effect of persistent nonadherence on relationships.

This study has some limitations. Since we used purposive sampling, the proportions may not accurately summarize the characteristics or opinions of the overall TB patient and supporter populations from which we selected. It is based on a single setting—results may be different in different settings where culture, use of technology, resources, interpersonal dynamics, and other factors vary. The study only included patients who possess mobile phones and know how to use SMS texts; patients without phones or/and without knowledge of using SMS texts may have different views. This study is also based on self-reported responses about sensitive topics, which may be vulnerable to social desirability bias. Importantly, several of our findings reflect views of an anticipated intervention without the benefit of experience of the intervention. Experiences with the intervention will be assessed in an upcoming trial (NCT03800888). This study also has a number of strengths. First, the achievement of theme saturation suggests the sample was adequate. The study highlights potential ways in which mobile phones could be used to promote TB medication. Our findings are important for understanding the potential acceptability of the use of SMS texts combined with electronic monitoring to support TB medication adherence, which may potentially be an attractive alternative to DOT. Additionally, these findings can inform ideal study design for future mobile health-based and other related interventions.

## 5. Conclusions

Mobile telephones could provide alternative approaches to providing social support for TB medication adherence especially where patients do not stay close to their social supporters. This could relieve transport and time burdens associated with traditional face-to-face delivery of support. This advantage may be particularly useful in resource-limited settings where mobile phones are widely adopted and other support is difficult to obtain. Further efforts should focus on optimized designs of mobile phone-based applications for providing social support to TB patients and training of TB patients and social supporters to match their expectations.

## Figures and Tables

**Table 1:** Sociodemographic and health characteristics of study participants.

Characteristic	TB patients (*n* = 35)	Social supporters (*n* = 15)
Female	15 (42.9%)	9 (60.0%)
Median age in years	32.0	37.0
Able to read English	27 (77.1%)	10 (66.7%)
Able to read Runyankole	34 (97.1%)	14 (93.3%)
Had regular income	18 (51.4%)	8 (53.3%)
Worried about food security	20 (57.1%)	8 (53.3%)
Living with HIV	15 (42.9%)	1 (6.7%)
Mean (standard deviation) of TB treatment period completed (months)	3.3 (1.5)	—

**Table 2:** Survey results from patients.

Current social support received by patients^∗^	
Reminders/encouragement to take medication	24 (69%)
Transport to clinic	21 (60%)
Food and drinks	20 (57%)
Help with chores	20 (57%)
Assistance with child care	10 (29%)
Counselling (e.g., encouragement to be positive about having TB)	5 (14%)
Money for other needs	3 (9%)
Adequacy of the social support received	
Social support enough	14 (40%)
Social support not enough	21 (60%)
Preference of notifications of social supporter	
Preferred notifications that instruct how the social supporter should help	15 (43%)
Preferred notifications that do not instruct how the social supporter should help	20 (57%)

^∗^Participants could select multiple responses.

## Data Availability

The data used to support the findings of this study are available from the corresponding author upon request.
